# 

**Published:** 1892-09

**Authors:** 


**Died.**—In Ridgetown, Ontario, Canada, Thursday, August 25, 1892, Dr. T. W. Schlenker, aged 24 years and 11 months. Dr. Schlenker spent last winter in Austin, in the hope of recovering his lost health. He was a victim of phthisis. Dr. Schlenker was a bright young physician, and had he lived he would have made his mark in the world.

Married—Aug. 18th ult., at Sulphur Springs, Texas, Dr. E. P. Becton to Mrs. Josephine Morris, both of that city.

The JOURNAL extends its cordial congratulations to the worthy and gifted disciple of Galen and Esculapius. He deserves a good wife, and being a man of sound and mature judgment, we are sure he has selected such. David of old took two virgins to "warm him up." Dr. Becton is always "warmed" up and can impart warmth. Its a blessing to hear him warm up in a speech. The Doctor has raised a family and has one son a doctor and one son-in-law in the same line, yet he is not an old man by any means. Age is not counted by years; he is yet young in heart and in sentiment and in full vigor of mental strength. Oliver Wendell Holmes boasted that he was "80 years young." So with our friend Becton, he is "—— years young."

